# Effect of wearing particulate respirators on physiological changes of healthcare workers in isolation room

**DOI:** 10.1017/ash.2025.133

**Published:** 2025-09-03

**Authors:** Titi Sundari, Vivi Setiawaty, Nina Mariana, Aninda Dinar Widiantari, Pompini Agustina Sitompul, Hotmarida Silalahi, Tri Diani Agustuti, Yustina Melandari, Farida Murtiani, Rina Yuliaty

**Affiliations:** Sulianti Saroso Infectious Disease Hospital

**Keywords:** Healthcare Workers, Quantitative Fit Test, Personal Protective Equipment

## Abstract

**Introduction:** The use of Personal Protective Equipment (PPE) for healthcare workers must be addressed, especially for procedures that generate aerosols. A standard N95, FFP2 or FFP3 particulate respirator mask is strongly recommended. WHO suggests using a particulate respirator for a maximum of six hours to avoid increasing oxygen debt, fatigue, CO2 levels, and nasal resistance. **Methods:** This study was observational, using a cross-sectional method conducted from February to April 2022. Participants were healthcare workers (HCWs), including doctors, nurses, and other HCWs who worked in ward of Mawar 1 Isolation Rooms. As screening, the participants underwent a Quantitative Fit Test with PortaCount® Respirator Fit Tester 8038, using particulate masks such as 3M 1870, 3M Vflex 9105, Dreamcan ME01LK, Dreamcan ME0 12.5, and RespoKare that are available in the hospital, while bending over, talking, head side to side, and head up and down. While doing the movement, the Fit Test Score had to reach ≥100. Then, we measured heart rate, oxygen saturation, and respiration rate before they entered and left the isolation rooms. **Result:** Thirty-one HCWs passed the screening test. One HCW could fit to more than one respirator. Sixteen (41,03%) HCWs fit to 3M Vflex 9105, 10 (25,64%) HCWs fit to Dreamcan ME01LK, 6 (15,38%)

HCWs fit to RespoKare, 4 (10,26%) fit to 3M 1870 and 3 (7,69%) fit to Dreamcan ME0 12.5. HCWs served in the isolation room for 74,06 ± 28,18 (35-150) minutes. We found a significant difference in heart and respiration rates before entering and after leaving the isolation room (p<0.05). In contradiction, the study showed no difference in O2 saturation (p=0,06).

